# Research Advances in Multiple Embryos and Apomixis in Rice (*Oryza sativa* L.)

**DOI:** 10.3390/ijms26157257

**Published:** 2025-07-27

**Authors:** Junhao Dan, Wuhua Long, Mudan Qiu, Longhui Zhang, Chaoxin Wu, Xue Jiang, Shengyan Fang, Susong Zhu, Huafeng Deng

**Affiliations:** 1 Guizhou Rice Research Institute, Guizhou Academy of Agricultural Sciences, Guiyang 550006, China; danjunhao0315@163.com (J.D.); mnslong@126.com (W.L.); wuchaoxin1995@163.com (C.W.); jurly55@163.com (X.J.); 18786154043@163.com (S.F.); 2 Ministry of Agriculture and Rural Affairs Key Laboratory of Crop Genetic Resources and Germplasm Innovation in Karst Region, Guiyang 550006, China; 3 College of Life and Environmental Science, Hunan University of Arts and Science, Changde 415000, China; 15580812842@126.com; 4 State Key Laboratory of Hybrid Rice, Hunan Hybrid Rice Research Center, Changsha 410125, China; zhanglh0623@163.com; 5 Institute of Crop Germplasm Resources, Guizhou Academy of Agricultural Sciences, Guiyang 550006, China

**Keywords:** synthetic apomixis, hybrid rice breeding, polyembryony

## Abstract

A typical seed of rice (*Oryza sativa* L.) gives rise to a single seedling. In contrast, seeds from multiple embryos may develop into two or more seedlings, one of which is generated via sexual reproduction, while the others are likely to originate through apomictic pathways. Therefore, the occurrence of multiple embryos is often considered a hallmark of apomixis in rice. Apomixis refers to an asexual reproductive strategy wherein unreduced gametes form through modified meiosis (apomeiosis) without fertilization, thereby generating clonal offspring generally genetically identical to the maternal plant. This process is of great relevance in fixing heterosis in hybrid rice breeding. This review discusses the origin, frequency, genetic regulation, and candidate genes related to multiple embryos in rice and provides a systematic summary of the latest research advances in rice apomixis. The insights presented in this study provide a theoretical foundation for the application of apomixis in rice breeding.

## 1. Introduction

Polyembryony refers to the occurrence of two or more embryos within a single seed. These embryos share a common endosperm and ultimately develop into multiple-embryo seedlings [1]. This phenomenon has been reported in a wide range of plant species, including *Citrus* species [2,3], *Coffea* [4], *Zanthoxylum* [5], *Melastoma sanguineum* [6], and *Oryza sativa* [7]. Among them, the polyembryony phenomenon in rice holds significant importance in the realm of agriculture. This trait can potentially lead to increased crop yields. Certain polyembryonic rice lines have been reported to produce multiple seedlings from a single grain [7,8]. If harnessed effectively, this could mean more plants per unit of seeds sown, optimizing land use and potentially increasing overall rice production.

**Figure 1:**
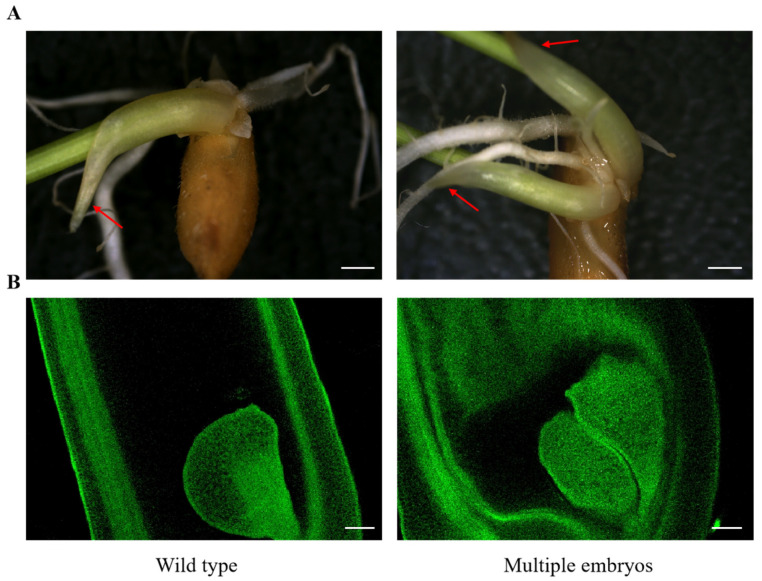
Multiple embryos in rice. (**A**) Morphology of multiple embryos. At 5 days after germination, wild-type seeds produced only one seedling exhibiting a single coleoptile (red arrow) and mesocotyl, whereas polyembryonic seeds generated multiple seedlings, each possessing an independent coleoptile (red arrow) and mesocotyl. Scale bar: 1 mm. (**B**) Embryo sac observation in multiple embryos; 72 h after plant flowering, a single pear-shaped embryo was observed at the micropylar end of the embryo sac in the wild type, while multiple pear-shaped embryos appeared at the micropylar end in polyembryonic plants. Scale bar: 100 μm. Left, wild type; right, multiple embryos.

In plants, polyembryony is often associated with apomixis and is widely regarded as one of its typical features [6]. In early studies conducted in China, research on rice apomixis primarily focused on identifying naturally occurring multiple-embryo materials and screening for germplasms with apomictic potential [9]. Compared to wild-type rice, seeds from multiple-embryo rice lines frequently produce two or more seedlings upon germination, with multiple embryos typically observed at the micropylar end of the embryo sac (Figure 1).

Apomixis refers to an asexual reproductive mode in plants whereby seeds are produced without fertilization (or pseudo-fertilization), typically involving the formation of unreduced gametes [10]. Unlike sexual reproduction, the meiotic process is often modified (apomeiosis) [11,12]. The resulting progeny generally retain the genetic traits of the maternal plant [13]. In apomictic plants, seeds are formed without fertilization, and the resulting progeny retain the desirable genetic traits of the maternal plant [14,15]. Accordingly, it has been proposed that apomixis could serve as an effective strategy for fixing heterosis in F_1_ hybrid progeny [16]. Longping Yuan put forward a strategic vision to shift hybrid rice breeding from the traditional ‘three-line’ system to a ‘one-line’ system, in which apomixis is employed to fix heterosis in F_1_ hybrids of hybrid rice [17]. This review summarizes the current progress on multiple embryos and apomixis in rice, aiming to provide a theoretical basis for the fixation of heterosis in hybrid rice breeding.

## 2. Multiple Embryos in Rice

### 2.1. Origins and Classification of Multiple Embryos

Sexual reproduction in rice occurs through double fertilization: one sperm cell fuses with the central cell to form endosperm, while the other combines with the egg cell to generate a zygote, which subsequently develops into a single embryo [18,19]. Chinese researchers initiated systematic screening and characterization of multiple-embryo rice as early as 1979, aiming to acquire germplasm resources exhibiting apomictic traits [9]. The primary origins of multiple-embryo rice include (1) the formation of multiple embryo-like structures from egg cell-like cells within the embryo sac [20]; (2) longitudinal division of the zygote resulting in multiple embryos [21,22]; (3) embryo initiation from synergid cells either before or after fertilization [8,23,24,25]; and (4) somatic cell-derived adventitious embryos generated via sporophytic apomixis [26,27].

Based on the origin of multiple embryos in rice, they can be classified into three categories: true polyembryony, false polyembryony, and adventitious embryony [28]. True polyembryony refers to the formation of multiple embryos from a single embryo sac, in which embryos originate from female gametophytic cells other than the egg cell through autonomous development. False polyembryony denotes multiple embryos arising from distinct embryo sacs, with each sac having a single embryo. Adventitious embryony involves somatic cells within the ovule differentiating into embryos that coexist with sexual embryos [28]. Due to the diverse origins of multiple embryos in rice, the resulting seedlings often exhibit variation in chromosome ploidy. In the case of twin-embryo rice, the predominant ploidy combinations include n-2n, 2n-2n, and 2n-3n [25]. Based on seedling ploidy, multiple-embryo rice is classified into haploid, diploid, and polyploid types, each originating from distinct cellular or tissue progenitors [8].

Haploid polyembryony is derived from unfertilized egg cells, synergid cells, or individual antipodal cells. Diploid polyembryony arises from fertilized egg cells, fertilized synergid cells, or somatic cells. Polyploid polyembryony is typically generated from antipodal cell clusters or central cells [8]. In addition, based on anatomical structure, multiple-embryo rice seedlings can be classified into complete and incomplete types. As shown in Figure 1, complete multiple-embryo seedlings are characterized by each individual seedling possessing an independent coleoptile and mesocotyl, indicating that each seedling has the ability to develop independently. In contrast, incomplete multiple-embryo seedlings either share both coleoptile and mesocotyl or share only the mesocotyl while maintaining separate coleoptiles [29].

### 2.2. Frequency and Influencing Factors of Multiple Embryos

#### 2.2.1. Frequency of Multiple Embryos

With in-depth investigations into multiple embryos in rice, researchers have progressively documented the frequency of multiple embryos and associated influencing factors. As shown in Table 1A, Li et al. [30] demonstrated that the frequency of twin embryos in the specific rice lines API, APII, APIII, and APIV was 16.1%, 23.4%, 32.4%, and 5.0%, respectively. Sun et al. [31] further investigated the types and frequency of multiple embryos in APIII and APIV and reported that APIII exhibited an 11.8% frequency of cleavage embryos, whereas APIV showed a 10.3% frequency of adventitious embryos. Through line selection, Guan et al. [27] obtained 55.0% and 30.0% twin embryos in APIII and APIV, respectively, demonstrating that artificial selection can significantly enhance the frequency of multiple embryos. Xiao [32] demonstrated that the twin-embryo frequencies in W3338 and W255 ranged from 15.6% to 36.4% and from 5.6% to 11.0%, respectively. Jiang [21] observed that the twin-embryo frequency in line W338-986 ranged from 13.3% to 43.5%, and that progeny derived from twin-embryo seedlings exhibited significantly higher twin-embryo frequencies than those derived from single-embryo seedlings. Research on multiple embryos in rice has extended beyond naturally selected diploid materials to include polyploid germplasm. As shown in Table 1B, Dai et al. [33] obtained twin-embryo frequencies ranging from 9.1% to 12.7% in the progeny of the autotetraploid line ‘IR36-Shuang’. The autotetraploid lines ASDR05-01 and ASDR05-02 exhibited twin-embryo frequencies of 9.8% and 3.4%, respectively [34]. The autotetraploid line D07-04-01 displayed a twin-embryo frequency of 1.3% [35]. These findings demonstrate that polyploid rice materials also possess the capacity to generate multiple embryos.

The advancement of genome editing technologies has enabled the development of rice mutants exhibiting multiple embryos through CRISPR/Cas9, which has led to extensive subsequent investigations. As shown in Table 1C, Puri et al. [7] obtained the fertile mutant *OsPE* exhibiting multiple embryos in the rice cultivar Basmati 370, through T-DNA/DS insertion. In the self-pollinated progeny of *OsPE* lines, the frequency of multiple embryos ranged from 9.8% to 21.0%, while offspring derived from single-embryo plants also exhibited multiple embryos at a frequency of 15.9–19.7%. Paul et al. [22] employed laser confocal microscopy to investigate the embryo sac structures of *OsPE* mutants at 2–5 days post-flowering and reported frequencies of multiple embryos ranging from 49.5% to 59.2% within the sacs. When integrated into prior studies, the observed frequency of multiple embryos (15.9–19.7%) was found to be significantly lower than embryonic frequencies within the sacs, likely due to intra-sac competition that hindered full seedling development. Beyond *OsPE* mutants, Xia et al. [36] achieved frequencies of twin embryos of 0.1% (T_1_) and 0.5% (T_2_) in hybrid rice 9You 418 by expressing the embryogenesis gene *AtWUS* under the somatic ovule-specific promoter *Os02g51090*, resulting in diploid (fertilized ovule-derived) and haploid (unfertilized gametophyte-derived) plants.

Additionally, Dan et al. [37] engineered the meiosis-related genes *PAIR1*, *OsREC8*, and *OsOSD1* in hybrid rice Yongyou 4949, generating *MiMe* mutants. The combination of *MiMe* with an embryogenesis cassette (*pAtDD45*:*BBM1*) achieved a high frequency of multiple embryos (61.0%). Subsequent integration of a fused promoter cassette (*pAtMYB98*_*pAtDD1*_*pOsECA1-like1*:*WUS*) produced multiple embryos at a frequency of 44.7%. This strategy resulted in the formation of twin embryos (2n/2n, 2n/4n, or 4n/4n) and triple embryos (4n/2n/2n or 2n/2n/2n), where diploid embryos originated through apomixis and tetraploids developed from zygote-derived embryos following fertilization. Ren et al. [38] co-expressed the high-efficiency enhancer *OsWOC9A* and the embryo-autonomous embryogenesis gene *OsBBM1* in egg cells, resulting in a twin-embryo frequency of 44.6% in the conventional rice cultivar Kitaake. The ploidy of twin embryos was either n/n or n/2n, with haploids formed as apomictic clones and diploids arising through sexual reproduction from fertilized egg cells (Table 1C).

#### 2.2.2. Influencing Factors of Multiple Embryos

The frequency of multiple embryos in rice is influenced by various factors, including modifier genes [30], temperature [34], seed husk removal [34], and pollination timing [39]. According to Xiao [32], the frequency of twin embryos is associated with the number of minor-effect genes involved. Hu established five temperature gradients ranging from 20 °C to 40 °C and found that the multiple-embryo frequency peaked when seeds were germinated at 30 °C. In addition, removing the seed husk increased the multiple-embryo frequency by 36.0–44.1% [34]. It has also been reported that delayed pollination promotes the occurrence of multiple embryos by stimulating the development of embryos other than the zygotic embryo, thereby increasing the frequency of multiple embryos [39].

### 2.3. Genetic Mechanisms of Multiple Embryos

Researchers have investigated the genetic mechanisms underlying the multiple-embryo trait in rice through a series of hybridization experiments. Li et al. [30] crossed lines API-APIV with a dominant single-embryo purple rice line and observed twin embryos in both F_1_ and F_2_ generations of the direct crosses, whereas no twin-embryo individuals were detected in the reciprocal crosses. Based on these findings, they proposed that the twin-embryo trait is governed by two pairs of recessive genes and may be influenced by maternal inheritance. Using the same materials (API and APIV), Luo et al. [39] conducted crosses with normal single-embryo lines and concluded that the twin-embryo trait is recessive and controlled by a single major-effect gene, with variation in modifier factors among different lines. Moreover, a higher frequency of twin embryos was observed in F_1_ plants from direct crosses compared with reciprocal crosses, suggesting a cytoplasmic inheritance effect. In contrast, the occasional presence of twin embryos in some reciprocal crosses was attributed to pollen direct induction [39]. However, Xiao [32] demonstrated that the twin-embryo trait in lines W3338 and W255 is determined by nuclear inheritance. The twin-embryo trait in W3338 was found to be controlled by two pairs of recessive genes, which is consistent with the viewpoint of Li et al. [30], while in W255 it was controlled by a single pair of recessive genes, supporting the conclusions of Luo et al. [39]. These results indicate variability in the genetic mechanisms underlying different multiple-embryo rice lines. Additionally, Xiao [32] proposed a distinct hypothesis based on his observation that twin embryos were absent in the direct cross (W338/R527) but present in the reciprocal cross (R527/W338). This led to the suggestion that the expression of the twin-embryo trait may be influenced by cytoplasmic factors and maternal effects, although further verification is required.

In another study, Jiang et al. [21] conducted direct and reciprocal crosses between the twin-embryo rice line W3339-986 and single-embryo rice lines Minghui 63 and 98-951. It was found that the F_2_ generation from both direct and reciprocal crosses exhibited twin-embryos, with a ratio of twin-embryo to single-embryo seedlings close to 1:15, suggesting that the twin-embryo phenotype was controlled by two pairs of recessive genes. This result was broadly consistent with previous findings [30,32]. Guan et al. [27] conducted direct and reciprocal crosses between the rice lines APIII and APIV and purple rice. The F_1_ generations of both types of crosses exhibited multiple embryos, with reciprocal crosses displaying a higher frequency. These findings suggested that the multiple-embryo phenotype in rice is governed by dominant nuclear inheritance, controlled by dominant polygenes, as inferred from phenotypic observations of hybrid progenies. However, the absence of molecular validation (e.g., SSR/SNP genotyping) precludes definitive conclusions, especially given contradictory recessive inheritance models reported elsewhere [30,39]. To resolve such discrepancies, future studies must implement marker-assisted selection to distinguish true allelic dosage effects from maternal or cytoplasmic influences.

### 2.4. Genes of Multiple Embryos in Rice

With advancements in molecular breeding techniques, candidate genes involved in the regulation of the multiple-embryo phenotype in rice have been identified through genomic and transcriptomic approaches. Puri et al. [7] identified a candidate gene associated with the multiple-embryo phenotype in rice, designated as *OsPE*, which is located on chromosome 3. Currently, no homologous genes of *OsPE* have been identified in the rice genome, and no conserved domain was found in the protein encoded by this gene. Xiong et al. [40] conducted evolutionary and functional analyses of the *OsPE*-encoded protein, revealing that its functionally annotated homologs are primarily involved in regulating plant stress responses and cell division processes, rather than the regulation of the multiple-embryo phenotype. As a result, it was suggested that the regulatory role of *OsPE* in the multiple-embryo phenotype of rice remains to be confirmed. In addition, the expression levels of alternative splice variants of the *OsPE* gene were further analyzed in the same study. The splice variant *OsPEc* was found to play a critical role in *OsPE* function, and the formation of multiple embryos in rice may be jointly regulated by *OsPEa*, *OsPEb*, and *OsPEc*.

In another study, Dan et al. [37] used the egg cell-specific promoter *pAtDD45* to ectopically express the embryo-autonomous development gene *BBM1* in egg cells, producing 1.5–3.9% twin-embryo seedlings (2n/n). These findings indicated that the *BBM1* gene is a key regulatory factor in the induction of multiple embryos in rice [38]. Protein structural analysis indicated that *BBM1* recognized cis-elements in embryonic development-related genes through its AP2/ERF domains, thereby indirectly regulating downstream targets of the auxin pathway [41]. The transcriptional activation domain of *BBM1* likely facilitated chromatin opening via epigenetic reprogramming, though specific mechanisms required further validation.

## 3. Overview of Apomixis in Rice

### 3.1. Classification of Apomixis in Rice

Apomixis is a form of clonal reproduction via seeds, where offspring generally inherit the maternal genotype and avoid generating variation by bypassing sexual fusion and recombination [42,43]. Currently, more than 140 genera of angiosperms have been reported to exhibit apomixis [44]. Based on the formation mechanisms of diploid clonal embryos, plant apomixis is classified into two major types: gametophytic apomixis and sporophytic apomixis [45]. Gametophytic apomixis entails embryo development through unreduced embryo sacs, categorized into diplospory and apospory based on the origin of the megagametophyte. In diplospory, the unreduced embryo sac arises directly from the megaspore mother cell (MMC) via three alternative pathways: mitotic diplospory, where the MMC undergoes mitosis; complete circumvention of meiosis (*Antennaria* type); or restitutional meiosis involving the omission of meiosis I/II to produce a diploid restitution product (*Taraxacum* and *Ixeris* types) [46]. Conversely, apospory involves the development of an unreduced embryo sac from a diploid somatic aposporous initial cell (AIC) adjacent to the MMC, which typically suppresses or degrades the MMC and its derivatives through mitotic divisions, as documented in *Hieracium* and *Pennisetum* [47,48], though in some taxa like *Brachiaria*, aposporous and sexual embryo sacs may coexist within a single ovule [49]. Sporophytic apomixis refers to a reproductive process in which diploid somatic cells within the ovule autonomously develop into clonal embryos [45]. The location of clonal embryo formation is random, and no endosperm is produced during this process. The clonal embryo shares the endosperm with the zygotic embryo and co-develops within the same seed, resulting in the presence of multiple embryos. This phenomenon is referred to as polyembryony.

### 3.2. The Process of Apomixis in Rice

Owing to the promising potential of apomixis, comprehensive investigations into apomictic reproduction in rice have been initiated. In 1986, China launched the National High-tech Research and Development Program of China (the ‘863 Program’), in which ‘Research on Apomixis in Rice’ was designated as one of the initial major scientific projects, led by scientists including academician Longping Yuan [50]. Since the occurrence of multiple embryos in rice is generally considered indicative of apomixis [51], the identification and selection of multiple-embryo rice lines became a major focus during the early stages of apomixis research in China [9,51].

Researchers have employed methods such as artificial selection [27,31], radiation-induced mutagenesis [33], temperature control [34], and hormone application [51] to enhance the frequency of multiple embryos in rice. Concurrently, investigations into the genetic characteristics of multiple embryos and the embryonic developmental processes of multiple-embryo seedlings have been conducted to elucidate the underlying genetic mechanisms and the origin of apomictic embryos in rice [22]. However, all apomictic materials derived from multiple embryos were haploid, and no diploid individuals were identified [51]. Due to their dwarf stature, high sterility, and limited tillering capacity [52], these early haploid apomictic materials had limited practical application and could not effectively fix the heterosis of hybrid rice—i.e., they failed to stably preserve the superior traits (e.g., high yield, stress resistance) exhibited by hybrid rice in their offspring, preventing the retention of hybrid vigor across generations. In recent years, with advancements and applications in modern molecular biology techniques, key genes associated with apomixis have been successively identified and characterized. Artificial induction of apomixis in rice has emerged as a principal approach to fixing heterosis in hybrid rice, yielding breakthrough achievements. The artificial induction of apomixis requires two critical elements: (1) the meiosis of female and male gametes is converted into mitosis-like division, producing clonal gametes without genetic recombination and reduction division; (2) the meiotically unreduced female and male gametes develop autonomously into embryos without nuclear fusion; that is, the clonal female gametes independently form embryos [50,53].

### 3.3. Advances of Synthetic Apomixis in Rice

#### 3.3.1. Generation of MiMe Mutants

**Figure 2:**
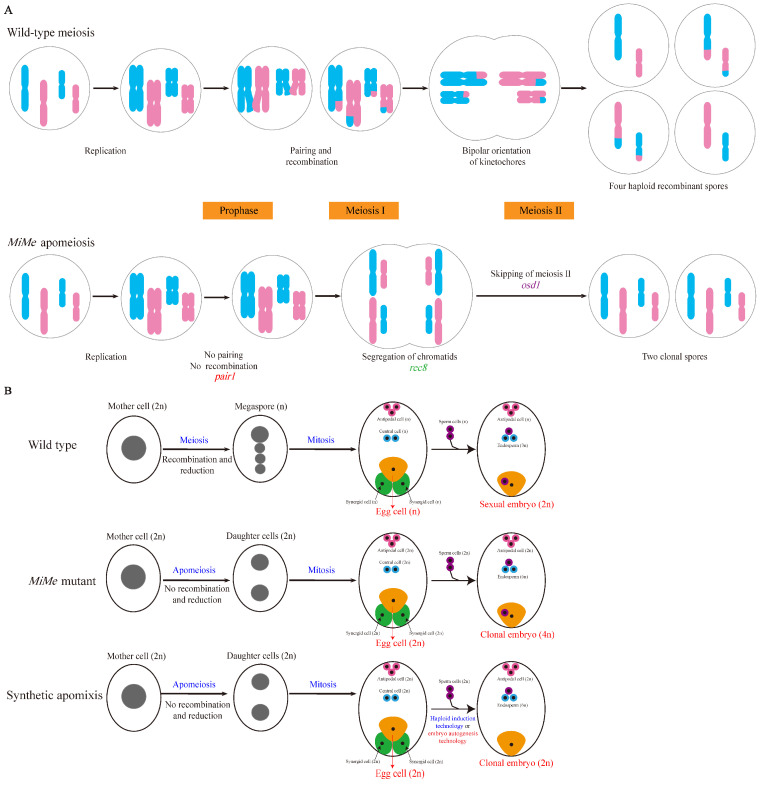
Schematic diagram synthetic apomixis in rice [55,63]. (**A**) *pair1*/*rec8*/*osd1* triple mutant meiosis in rice. The *MiMe* system turns meiosis into a mitosis-like process through three targeted mutations: (i) prevention of homologous chromosome pairing and recombination (*pair1*); (ii) induction of bipolar segregation of sister chromatids during meiosis I (*rec8*); (iii) skipping of meiosis II (*osd1*). The resulting spores are genetically identical to the maternal plant (apomeiosis). Mutation of genes marked in red, green, and purple leads to no pairing and no recombination, bipolar segregation of chromatids, and skipping of meiosis II, respectively. (**B**) Synthetic apomixis via *MiMe* integration. The combination of the *MiMe* with either haploid induction technology (e.g., *MTL* or *OsPLDα2* mutation) [56,57] or embryo autogenesis technology (e.g., ectopic expression of *BBM1* or *BBM4* in the egg cell) [58,60] enables the development of a clonal embryo.

Mitosis instead of meiosis is a prerequisite for synthetic apomixis in rice. Simultaneously mutating *AtSPO11-1*, *AtREC8*, and *OSD1* resulted in the replacement of meiosis with a mitosis-like division, thereby generating the *MiMe* mutant [54]. In 2016, Mieulet et al. [55] employed a pairwise crossing strategy to simultaneously knock out *PAIR1*, *OsREC8*, and *OSD1*, thereby establishing the *MiMe* system in rice through in vivo genetic editing (Figure 2A). Clonal gametes genetically identical to the maternal parent can be produced by *MiMe* mutants; however, progeny arising from normal double fertilization exhibits doubled ploidy levels [54,55]. To achieve synthetic apomixis in rice, the *MiMe* system must be further engineered with (1) haploid induction technology (e.g., by mutating *MTL* or *OsPLDα2*) [56,57], which leads to embryo formation under conditions of severe fragmentation of male gametes [58,59] or (2) ectopic expression of embryogenesis genes (e.g., *BBM1* or *BBM4*) [58,60], which enable autonomous development of the egg cell without fertilization [61,62]. Both strategies operate through synthetic genetic pathways in rice, allowing the generation of clonal plants devoid of paternal genetic material (Figure 2B) [55,63].

#### 3.3.2. Establishment of a Synthetic Apomixis System

**Figure 3:**
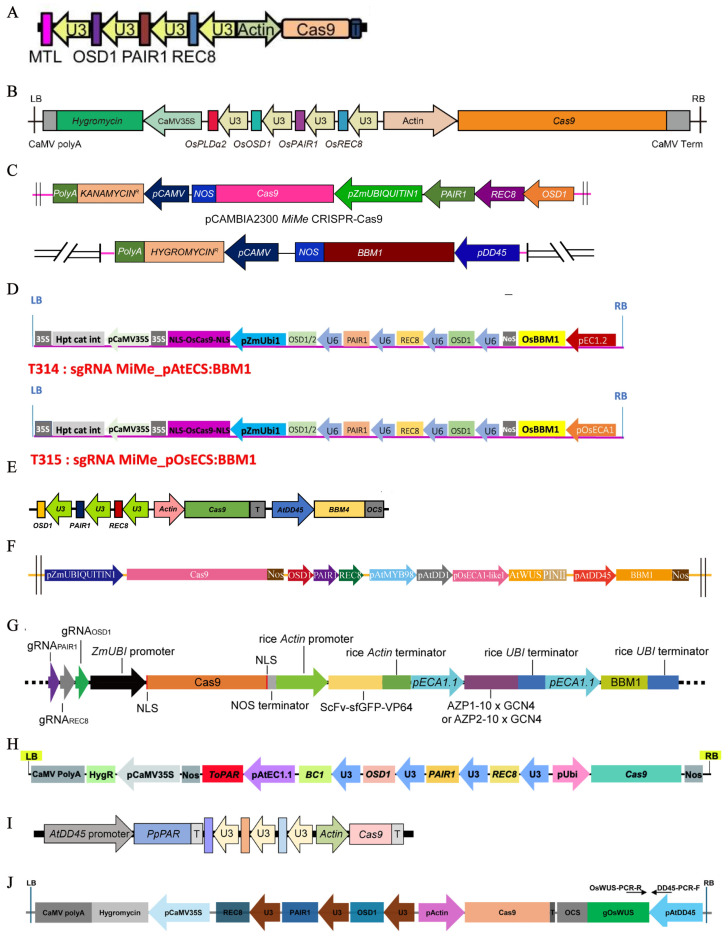
Schematic diagram of T-DNA insertion fragment [37,56,57,58,60,71,73,77,78,79]. (**A**–**J**) are all schematic diagrams based on the *MiMe* system (a T-DNA structure targeting *OsOSD1*, *PAIR1*, and *OsREC8*). (**A**) Schematic diagram of the T-DNA construct *MiMe*_*MTL*. (**B**) Schematic diagram of the T-DNA construct *MiMe*_*OsPLDα2*. (**C**) Schematic diagram of the T-DNA construct *MiMe*_*pAtDD45*:*BBM1*. (**D**) Schematic diagram of the T-DNA construct *MiMe*_*pECS*:*BBM1* (single step). (**E**) Schematic diagram of the T-DNA construct *MiMe*_*pAtDD45*:*BBM4*. (**F**) Schematic diagram of the T-DNA construct *MiMe*_*pAtMYB98*+*pAtDD1*+*pOsECA1-like1*:*AtWUS*_*pAtDD45*:*BBM1*. (**G**) Schematic diagram of the T-DNA construct *MiMe*-*ECA1*-*AZP2*_*pECA1.1*:*BBM1*. (**H**) Schematic diagram of the T-DNA construct *MiMe*_*pAtEC1.1*:*ToPAR*. (**I**) Schematic diagram of the T-DNA construct *MiMe*_*pAtDD45*:*PpPAR*. (**J**) Schematic diagram of the T-DNA construct *MiMe*_*pAtDD45*:*OsWUS*.

*MTL* is a phospholipase-encoding gene specifically expressed in sperm cells and has been shown to induce haploid formation at a frequency of 2.0–6.0% in rice [64]. In 2019, Wang et al. [56] combined the *MiMe* mutant with the haploid-inducing gene *MTL* and to develop apomictic lines in the hybrid rice cultivar Chunyou 84, designated as *Fix* (Figure 3A). The apomixis efficiency of *Fix* ranged from 4.7% to 9.5%, with a seed-setting rate of 3.7–5.2%. Liu et al. [65] propagated the *Fix* lines to the T_4_ generation. Through analysis of agronomic traits, genome, methylome, and transcriptome profiles from the T_1_ to T_4_ clonal generations, it was found that both apomictic characteristics and agronomic performance remained stable across generations, with clonal plant frequencies ranging from 3.0% to 4.3%. Xie et al. [66] simultaneously knocked out *OsSPO11-1*, *OsREC8*, *OsOSD1*, and *OsMATL* in the conventional rice cultivar Yandao 8, thereby generating an *AOP* mutant, enabling the transition from sexual reproduction to apomixis in rice. *PLD* plays a crucial role in plant apical growth and cell expansion and can induce haploid production at a frequency of 0.3–0.6% in rice [57,67]. In 2025, Hu et al. [57] combined the *MiMe* mutant with the haploid-inducing gene *OsPLDα2* (Figure 3B), successfully developing the *Fix4* in the hybrid rice cultivar Chunyou 84. The *Fix4* exhibited an apomictic efficiency of 0.8–1.2% and a seed-setting rate of 82.1–85.0%.

*BBM*-like genes encode transcription factors belonging to the AP2/ERF family. These genes are preferentially expressed in embryos and seeds, and ectopic expression of *BBM* has been shown to induce somatic embryogenesis [68,69]. *BBM1*, a member of the *BBM*-like gene family, has been found to be transcribed and translated in sperm cells prior to fertilization. After fertilization in rice, the *BBM1* gene continues to exhibit expression in the zygote through to the globular embryo stage [58,70]. In 2018, Khanday et al. [58] introduced ectopic expression of *BBM1* under the control of the egg cell-specific promoter *AtDD45* within a *MiMe* background (Figure 3C), resulting in successful induction of parthenogenesis in the egg cell. This approach yielded 11.1–29.2% clonal plants in the conventional rice cultivar Kitaake. In 2022, Vernet et al. [71] developed *AtECS:BBM1* and *OsECS:BBM1* expression cassettes within the *MiMe* background using a single step (Figure 3D), achieving over 95.0% clonal seeds at a high frequency in the hybrid rice cultivar BRS-CIRAD 302, with clonal plant seed-setting rates ranging from 27.0% to 35.5%. In addition to *BBM1*, three additional *BBM*-like genes—*BBM2*, *BBM3*, and *BBM4*—have been identified, each exhibiting distinct expression patterns in rice [58,72]. Wei et al. [60] ectopically express the embryogenesis gene *BBM4* under the *AtDD45* promoter in egg cells, in combination with the *MiMe* mutant (Figure 3E), resulting in 1.3–2.4% clonal seeds in the hybrid rice cultivar Chunyou 84, with a seed-setting rate of 80.9–82.0%. In 2024, Dan et al. [37] introduced *AtDD45*:*BBM1* together with the expression cassette *AtMYB98*_*AtDD1*_*OsECA1-like1*:*AtWUS* into a *MiMe* genetic background (Figure 3F). In the hybrid rice cultivar Yongyou 4949, this strategy achieved up to 98.2% clonal plants, with a maximum seed-setting rate of 83.7%. In 2025, Song et al. [73] achieved over 95.0% clonal seed production by combining *MiMe*-*ECA1*-*AZP2* with ectopic expression of the *BBM1* gene in the hybrid rice cultivar Yongyou 4949 (Figure 3G).

*PAR* encodes a K2-2 zinc finger protein containing an EAR domain. It originates from *Taraxacum officinale*, a species that exhibits apomictic traits and has been shown to induce autonomous embryo development in the unfertilized egg cell [74,75]. In the sexually reproducing plant *Lactuca sativa*, ectopic expression of the *PAR* gene under the control of the egg cell-specific promoter *AtEC1.1* has been demonstrated to induce parthenogenesis in the egg cell [76]. In 2023, Song et al. [77] ectopically expressed the parthenogenesis gene *ToPAR* in the egg cell using the egg cell-specific promoter *AtEC1.1* and combined this construct with the *MiMe* mutant (Figure 3H). As a result, clonal seeds were obtained at frequencies ranging from 42.9% to 67.7% in the hybrid rice cultivars Jiaheyou 7245 and Jiafengyou 2, with seed-setting rates ranging from 72.7% to 75.6%. The *PpPAR* gene exhibits highly conserved structural and expression patterns with the *ToPAR* gene, establishing it as a key candidate gene for parthenogenesis in hawkweed (*Pilosella piloselloides*) [76,77]. In 2025, Xiong et al. [78] achieved haploid production at a frequency of 0.5–1.5% through ectopic expression of *PpPAR* in the hybrid rice cultivar Chunyou 84. Further combining the *PpPAR* gene ectopic expression with the *MiMe* mutant resulted in the successful development of the apomictic *Fix5* line (Figure 3I). The *Fix5* demonstrated an apomictic efficiency of 21.2–84.6% and a seed-setting rate of 52.1–59.6%.

In addition, studies have shown that the endogenous rice gene *WUS* has also been shown to induce apomixis in rice [79]. The *OsWUS* gene encodes a transcription factor belonging to the HOMOBOX family that functions as a key regulator of stem cell differentiation by maintaining a balance between proliferation and differentiation [80,81]. In addition, it governs the fate of megaspore mother cells by regulating the expression of *WIH1* and *WIH2* [82,83,84]. In 2024, Huang et al. [79] ectopically expressed the embryogenesis-related gene *OsWUS* in the egg cell using the *AtDD45* promoter. When combined with the *MiMe* mutant, this strategy resulted in 0.5–21.7% clonal seeds, with seed-setting rates ranging from 72.0% to 85.2% (Figure 3J). In summary, apomixis in rice has achieved a significant breakthrough from concept to realization, heralding a new era for fixing heterosis in hybrid rice through apomictic reproduction (Table 2).

#### 3.3.3. Clonal Seed Sorting System

**Figure 4:**
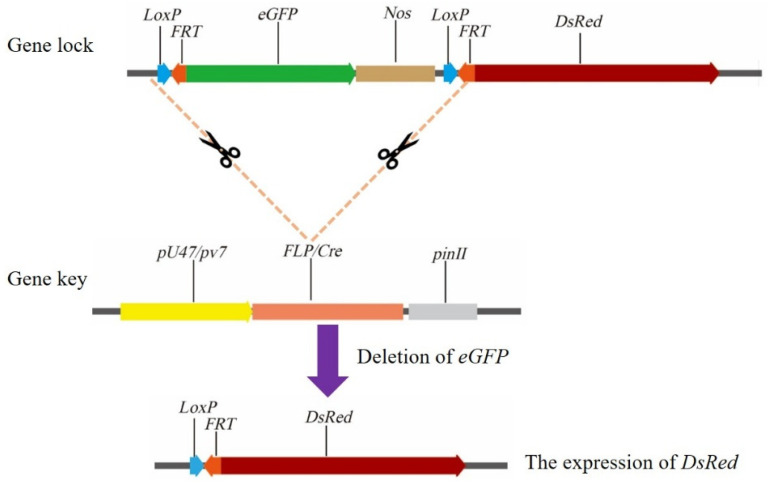
The principle of the gene switch system [86,87].

The clonal seed sorting system is a recombinase-based technology that employs pollen-specific *Cre*/*FLP* recombinases to excise the fluorescent marker between *loxP* + *FRT* sites, enabling the screening of clonal seeds via an alternative fluorescent marker [85,86,87]. In this system, *LoxP* + *FRT*-*eGFP*-*LoxP* + *FRT* is the ‘gene lock’, whereas *Cre*/*FLP* recombinases serve as the ‘gene key’. Red fluorescence is detected when the ‘gene key’ specifically recognizes the *LoxP* + *FRT* sites and excises the ‘gene lock’. In contrast, if excision does not occur, green fluorescence is observed (Figure 4). This genetic switch system achieved an unlocking efficiency of 89.7% in *Escherichia coli* [86]. Subsequently, Zhan et al. [87] used the pollen-specific promoters *pG47*/*pv4* to drive expression of the *Cre* recombinase in the hybrid rice cultivars Yongyou 4949 and Yongyou 2640. This enabled differentiation between sexual seeds, which exhibited red fluorescence at the shoot apical meristem, and clonal seeds, which lacked fluorescence at this site. The sorting efficiency reached 80.2–88.9%, providing a promising solution to the challenge of simultaneously achieving high clonal seed induction rates and high seed-setting rates in clonal plants.

## 4. Conclusions and Perspectives

The phenotype of multiple embryos in rice exhibits a certain degree of complexity. First, progeny derived from multiple-embryo rice lines may produce both multiple-embryo and single-embryo seedlings, while even single-embryo offspring retain the capacity to generate both seedling types. Second, the origin of the multiple seedlings within a single multiple-embryo seed may vary, originating from either sexual reproduction or apomictic development. In summary, identifying apomixis-related genetic resources from naturally occurring multiple-embryo rice germplasm presents considerable challenges. From a breeding perspective, stable diploid apomictic embryos derived from multiple-embryo traits enable the fixation of heterosis in hybrid rice, significantly reducing hybrid seed production costs. Concurrently, the coexistence of sexual and apomictic embryos within a single seed provides an efficient platform for rapid screening of elite genotypes, accelerating the development of stable high-yielding lines. In terms of production applications, multiple-embryo seeds containing viable diploid clonal embryos increase effective seedling density per unit area, potentially reducing seeding rates. Critically, these clonal embryos inherit parental traits such as disease resistance and high yield, ensuring stable productivity across diverse environments.

Recently, the *Os02g51090*:*AtWUS* vector was constructed using gene-editing techniques, resulting in twin-embryo rice materials with n-2n ploidy combinations. In this system, the haploid seedling originates from gametophytic apomixis, but, due to its lower competitiveness compared to the diploid seedling, seldom survives—leading to a low frequency of twin-embryo seedlings [36]. It is proposed that this strategy could be integrated into the *MiMe* mutant by constructing an *sgMiMe*_–_*Os02g51090*:*AtWUS* vector and introducing it into elite hybrid rice cultivars. The resulting progeny may generate twin embryos with 2n-4n ploidy combinations, with the diploid representing a clonal plant. Compared with the sexually derived tetraploid, the diploid is expected to exhibit a stronger competitive advantage. This approach offers a novel perspective for exploring apomixis in rice through the lens of the multiple-embryo phenotype.

**Figure 5:**
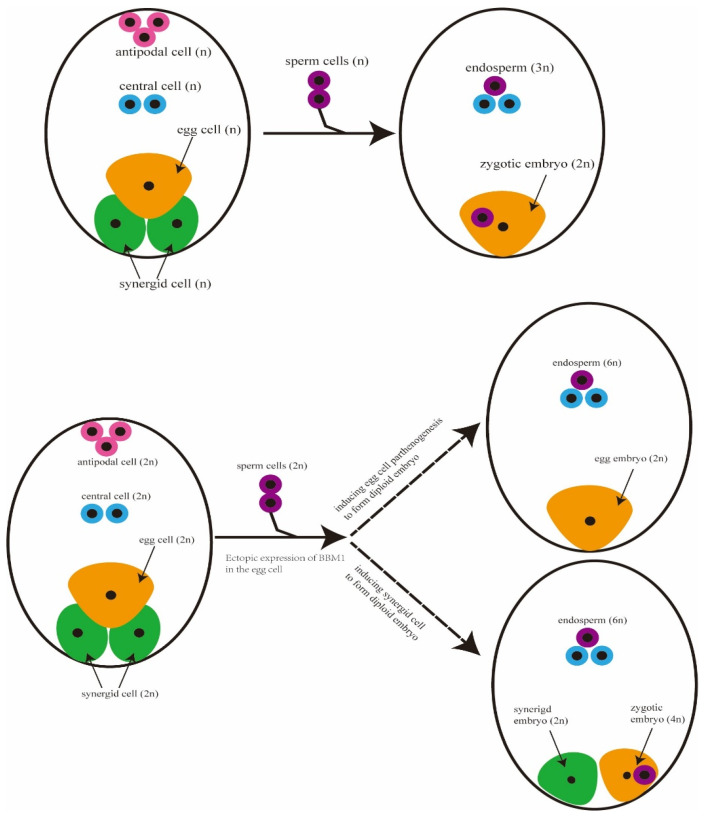
Schematic diagram of multiple embryos (2n/4n). Top: wild-type rice, where haploid gametes are normally fertilized to form a zygote embryo (2n) and an endosperm (3n). Bottom: multiple embryos. Diploid clonal gametes are generated and allow the egg cell to form clonal embryo (2n, parthenogenesis); when the egg cell is normally fertilized to form a zygotic embryo (4n), the synergetic cell develops autonomously to form a synergistic embryo (2n, apogamy), and the zygotic embryo and the synergistic embryo share the endosperm (6n).

Dan et al. [37] combined *MiMe* with ectopic expression of *BBM1* in the egg cell and obtained up to 61.0% multiple-embryo seedlings. Given that the ploidy combinations observed in these seedlings included a 2n/4n configuration, it was hypothesized that the egg cell may follow two distinct reproductive pathways. One involves the production of an asexual embryo via parthenogenesis, wherein the sperm fuses with the central cell to form the endosperm, ultimately generating an asexual embryo-derived seed. The other involves normal fertilization of the egg cell by sperm to form a zygotic embryo, while the synergid cell autonomously develops into an asexual embryo via apogamy. In this case, the zygotic and asexual embryos share a common endosperm, resulting in the formation of a twin-embryo seed (Figure 5). To elucidate the underlying mechanisms of multiple embryos, Peha et al. [88] proposed two models regarding the origin of twin-embryo structures: (1) Dizygotic Model—under the influence of *BBM1*, the unfertilized egg cell transforms into an apomictic zygote, and one of the synergid cells is reprogrammed into a functional egg cell. (2) Monozygotic Model—following the parthenogenesis of the egg cell, the resulting apomictic zygote retains pluripotency after initial division, giving rise to two apomictic zygotes. These two models may function independently but could also act in combination. However, the occurrence of multiple embryos will hinder the commercial application of materials developed for fixing heterosis. Based on the aforementioned two models, the timing and level of *BBM1* expression could be fine-tuned to reduce the phenotype of multiple embryos. For example, a cell-specific promoter could be used to drive *BBM1* expression after the degeneration of synergid cells. Alternatively, reducing the expression level of *BBM1* in egg cell may limit the pluripotency of the apomictic zygote following division, thereby decreasing the incidence of multiple embryos in rice [88].

Current studies have demonstrated the feasibility of fixing heterosis in hybrid rice through synthetic apomixis. However, the simultaneous achievement of both high apomictic efficiency and high seed-setting rates for clonal plants remains challenging, limiting its industrial application. This limitation manifests in two key ways: (1) Apomictic materials capable of producing clonal seeds at a high frequency often exhibit low seed-setting rates. Vernet et al. [71] achieved up to 95.0% clonal seeds in rice using a single-step strategy (*sgMiMe*_–_*pOsECS*:*BBM1*); however, the seed-setting rate of clonal plants was only 27.0–35.5%, significantly lower than that of wild-type plants (average of 44.4%). The researchers attributed this fertility reduction to the incomplete penetrance of *MiMe* during female meiosis. They proposed that integrating meiotic cell cycle regulators like *TAM* or *TDM* could enhance the expression efficiency of the *OsOSD1* gene. (2) Conversely, apomictic materials with high seed-setting rates often produce clonal seeds at a low frequency. Wei et al. [60], using the *sgMiMe*_–_*pAtDD45*:*BBM4* system, achieved a high seed-setting rate of 80.9–82.0% in clonal plants. However, due to the low penetrance of *BBM4*, this strategy yielded only 1.3–2.4% clonal seeds. Similarly, Hu et al. [57] constructed a four-gene knockout vector (*sgMiMe*_–_*OsPLDα2*) and obtained clonal plant with a high seed-setting rate of 82.1–85.0%. Nevertheless, the apomictic efficiency was merely 0.8–1.2%.

Research indicates that the parthenogenesis-enhancing factor *OsWOC9A* can significantly increase parthenogenesis efficiency from 29.2% to 91.0%, representing a 4- to 15-fold enhancement compared to the expression of *OsBBM1* alone [39]. To address these limitations and achieve a breakthrough in rice apomixis (from feasibility to practical application), we propose exploring two complementary strategies: (1) For lines already exhibiting high clonal seed production, genes could be introduced that regulate endosperm development, such as *FIS*-like genes or *FIE*-like genes [89], to modulate endosperm cell proliferation and thereby increase the seed-setting rate of clonal plants. (2) For lines already exhibiting high seed-setting rates, we recommend the integration of the parthenogenesis-enhancing factor *OsWOC9A* with ectopic expression of *BBM1* and knockout of haploid-inducing genes. This could involve constructing vectors such as *sgMiMe*_–_*pOsECS*:*OsWOC9A*_–_*BBM1*_–_*BBM4*, *sgMiMe*_–_*pOsECS*:*OsWOC9A*_–_*BBM1*_–_*OsPLDα2*, and *sgMiMe*_–_*pOsECS*:*OsWOC9A*_–_*BBM1*_–_*MTL*. These constructs could be introduced into elite hybrid rice cultivars in a single step, aiming to develop apomictic materials exhibiting both high clonal seed induction rates and high seed-setting rates. Simultaneously, a dual-pronged approach to gene discovery is warranted. On the one hand, we should screen for endogenous rice genes associated with embryo development and somatic cell proliferation. On the other hand, we must identify exogenous genes known to confer apomictic traits in other species. The ectopic expression of these identified endogenous or exogenous genes specifically within the egg cell should be investigated to assess their potential for inducing apomixis in rice.

## Figures and Tables

**Table 1:** (**A**) Frequency of multiple embryos in different rice varieties (naturally selected diploids). (**B**) Frequency of multiple embryos in different rice varieties (artificially induced polyploids). (**C**) Frequency of multiple embryos in gene-edited rice mutants.

Rice Cultivar	Multiple Embryos/%	Ref.
(**A**) Frequency of multiple embryos in different rice varieties (naturally selected diploids)		
API	16.1	[30]
APII	23.4	[30]
APIII	32.4, 55.0	[27,30]
APIV	5.0, 30.0	[27,30]
W3338	36.4	[32]
W255	11.0	[32]
W338-986	43.5	[21]
(**B**) Frequency of multiple embryos in different rice varieties (artificially induced polyploids)		
IR36-Shuang	12.7	[33]
ASDR05-01	9.8	[34]
ASDR05-02	3.4	[34]
D07-04-01	1.3	[35]
(**C**) Frequency of multiple embryos in gene-edited rice mutants		
*OsPE*	21.0	[7]
*Os02g51090*:*AtWUS*	0.5	[36]
*MiMe* + *pAtDD45*:*BBM1*	61.0	[37]
*MiMe* + *pAtMYB98*_*pAtDD1*_*pOsECA1-like1*:*WUS*	44.7	[37]
*pEC1.2*:*OsWOC9A + OsBBM1*	44.6	[38]

**Table 2:** Quantitative analysis of synthetic apomixis strategies in rice: clonal seed induction rates and seed-setting rates across experimental systems.

Method	Clonal Seed Induction Rate/%	Seed-Setting Rate/%	Year	Ref.
*MiMe* + *MTL*	4.7–9.5	3.7–5.2	2019	[56]
*MiMe* + *BBM1*	11.1–29.2	No data	2019	[58]
*MiMe* + *BBM1* (single step)	80.0–100.0	27.0–35.5	2022	[71]
*MiMe* + *BBM4*	1.3–2.4	80.9–82.0	2023	[60]
*MiMe* + *ToPAR*	42.9–67.7	72.7–75.6	2024	[77]
*MiMe* + *BBM1* + *AtWUS*	5.4–98.2	15.8–83.7	2024	[37]
*MiMe*-*ECA1*-*AZP2* + *BBM1*	1.7–100.0	63.8–87.7	2024	[73]
*MiMe* + *OsWUS*	0.5–21.7	72.0–85.2	2025	[79]
*MiMe* + *OsPLDα2*	0.8–1.2	82.1–85.0	2025	[57]
*MiMe* + *PpPAR*	21.2–84.6	52.1–59.6	2025	[78]

## Data Availability

No new data were created or analyzed in this study.

## Abbreviations

The following abbreviations are used in this manuscript:

PAIR1	HOMOLOGOUS PAIRING ABERRATION IN RICE MEIOSIS 1
REC8	RECOMBINATION8
OSD1	OMISSION OF SECOND DIVISION
MiMe	mitosis instead of meiosis
WOC9A	WUSCHEL-LIKE HOMEODOMAIN 9
DD45	DOWNREGULATED IN DETERMINANT INFERTILE1 45
BBM	BABY BOOM
SPO11-1	SPORULATION11-1
MTL	MATRILINEAL
Fix	Fixation of hybrids
PLD	Phospholipase D
AOP	Apomictic Offspring Producer
AP2/ERF	APETALA 2/ETHYLENE RESPONSE FACTOR
PAR	PARTHENOGENESIS
EAR	ethylene-responsive element-binding factor-associated amphiphilic repression
WUS	WUSCHEL
WIH1	WINDHOSE1
WIH2	WINDHOSE2
FIS	fertilization-independent seed
FIE	FERTILIZATION-INDEPENDENT ENDOSPERM
FLP	Flipase recombinase
